# Double jeopardy: How lower levels of support during COVID-19 exacerbated the relationship between loneliness and distress

**DOI:** 10.3389/fpubh.2022.976443

**Published:** 2022-08-26

**Authors:** Sarah V. Bentley, Tarli Young, Belén Álvarez, Jolanda Jetten, Catherine Haslam, Tegan Cruwys, Bruno Gabriel Salvador Casara, Charlie R. Crimston, Michael Dare, Octavia Ionescu, Henning Krug, Hema Preya Selvanathan, Porntida Tanjitpiyanond, Niklas K. Steffens, Zhechen Wang, Susilo Wibisono

**Affiliations:** ^1^School of Psychology, The University of Queensland, Brisbane, QLD, Australia; ^2^Research School of Psychology, The Australia National University, Canberra, ACT, Australia; ^3^University of Padua, Padova, Italy; ^4^Laboratoire Parisien de Psychologie Sociale, Université Paris 8 Vincennes, Saint Denis, France; ^5^Philipps University of Marburg, Marburg, Germany; ^6^School of Social Development and Public Policy, Fudan University, Shanghai, China; ^7^Department of Psychology, Universitas Islam Indonesia, Yogyakarta, Indonesia

**Keywords:** loneliness, COVID-19, social support, social identity, psychological distress

## Abstract

While the relationship between loneliness and psychological distress is well documented, the mechanisms underlying this relationship are less clear. One factor known to be related to loneliness as well as psychological distress, is social support, with some studies suggesting that support–both received and provided–can serve as a mechanism to reduce the distress associated with loneliness. In this paper we examine the mediating role of both aspects of support in the relationship between loneliness and psychological distress in the COVID-19 context. We used a multi-country dataset collected at two timepoints during the pandemic; the first during the early stages (*N* = 6,842, 11 countries) and the second collected for a subset of countries (*N* = 1,299, 3 countries) 3 months later. Across all eleven countries, results revealed significant positive associations between loneliness and distress. Furthermore, using longitudinal data, we investigated the directionality of this relationship and found that increased loneliness over time was associated with increased psychological distress. The data also showed that both feeling unsupported and feeling unable to provide support to others mediated this relationship. These findings point to the need to facilitate people's ability to draw effective social support and help others–particularly at times when social connectedness is threatened–as a way of alleviating the psychological distress that commonly presents with loneliness.

## Introduction

Loneliness is generally described as a negative experience, arising from the feeling that one's social needs are not met by one's social relationships (1–3). While loneliness is not a new phenomenon, it became highly salient during the COVID-19 pandemic; a time when social engagement was tangibly reduced by virtue of people needing to isolate at home to stop the spread of the virus (4). Over a period of approximately 2 years, people across the world worked from home more (5), traveled less (6), and engaged in significantly fewer social activities (7). For many people, feeling cut off from family, friends, and work colleagues resulted in increased levels of social isolation and loneliness (8–12). This was supported by data showing higher rates of loneliness for people living under lockdown orders (reducing social contact opportunities) than those living with no restrictions (8, 9, 13).

The impact of loneliness on a person's quality of life is significant, and is often associated with increased psychological distress, in the form of anxiety and depression (14, 15). These negative wellbeing effects also came to the fore when the COVID-19 pandemic hit, with data showing increased levels of reported distress during the pandemic (16). Furthermore, data collected showed that these effects were greater for vulnerable groups such as people on low incomes, those with pre-existing mental illness, or more generally, people with less social support (11, 13, 17–20).

Previous research has shown how social support can play a role in reducing loneliness, as well as in countering psychological distress (21–23). However, opportunities to both feel supported as well as to provide support for others were also diminished by the social restrictions put in place to manage COVID-19 (8, 9, 13). It is likely that this further exacerbated both loneliness and psychological distress during the pandemic (24–27). In a study which included samples from eleven countries taken during the pandemic, we examined the relationship between loneliness and psychological distress. In particular, we focused on social support–both received and provided–as a hypothesized mechanism through which loneliness influences psychological distress. Before elaborating on why social support provides an explanation for the relationship between loneliness and psychological distress, we will first step back to assess the social underpinnings of loneliness, and why–theoretically speaking–loneliness enhances psychological distress. We propose that the social identity approach provides a theoretical model from which to understand the relationships between social (dis)connection, loneliness, and psychological distress.

### Loneliness and psychological distress: A social identity approach

While the relationship between loneliness and psychological distress may seem intuitive–it is emotionally painful to feel lonely due to a sense of being socially disconnected–few theoretical frameworks have examined the question of why loneliness should enhance psychological distress. Here, we propose that the social identity approach–combining Social Identity Theory and Self-categorization Theory principles (28–31)–might help to theorize this relationship. The social identity approach describes how a person's sense of self is informed by their group memberships, and more specifically, the strength of identification with them (32–34). Tajfel [(35). p. 78] defined social identity as the part of a person's self-concept informed by group memberships and from which is derived “value and emotional significance”. Self-categorization theory was subsequently developed to provide a socio-cognitive account of the process of social identification. It describes how and when social identities are activated, and how the salience of group memberships affects the self (30, 31, 36, 37).

Research informed by the social identity approach has shown how group memberships (and the social identities that are derived from group memberships) affect people's self-esteem, belonging, meaning, sense of purpose, and efficacy (38–40). Given the central role of group memberships in how people think, feel, and behave (39–42), recently, social identity theorizing has been extended to focus on understanding the social processes that underlie health and wellbeing outcomes [the Social Identity Approach to Health, SIAH, (33, 43–45)].

This sub-discipline of social identity research describes how a sense of positive group membership is key to understanding a range of health outcomes (32, 46, 47). Referred to as the *Social Cure*, this perspective has demonstrated how social connection can improve feelings of personal control (39), satisfy global psychological needs (48), enhance resilience (49), alleviate depression (50), and even reduce post-retirement mortality rates (51). Findings from the social cure perspective suggest that it is both the process and strength of identification with groups that provides a base from which to access health-giving psychological resources. This relationship has been demonstrated empirically with a range of populations, from heart surgery patients (52) to Australian school students (53). Consistent with this perspective, large-scale epidemiological studies have demonstrated that a positive sense of social integration and support strongly predicts health outcomes, including longevity (54–56).

As much as social connection is good for health, social disconneciton is a risk to health. To understand just how important social connectedness is for humans, consider situations where opportunities for social interaction are lacking, for instance, conditions of ill-health, old age, or social restrictions. Considerable evidence suggests that being cut-off from social interaction with groups that matter to people can have a profoundly negative effect on people's resilience, health, and wellbeing, and can even lead to early death [for a review, see (32, 45)]. Social isolation represents a health hazard because people are no longer able to reap the psychological benefits of group membership. Consistent with this reasoning, inadequate social connection is known to lead to an increased sense of being lonely (54), the most common impact of which is increased psychological distress (14, 15, 57, 58). In order to understand the basis of this relationship, social identity research has explored the types of resources unlocked through membership of groups, such as social support (59, 60).

### A social identity analysis of social support

Defined as “various forms of aid and assistance supplied by family members, friends, neighbors, and others” [(61). p. 435], social support has long been recognized as an important public health factor (62, 63), with data showing that a perceived lack of social support can be associated with increased loneliness (22, 23) as well as with heightened levels of distress, psychological maladjustment, and physical illness (21, 64, 65). It is still not clear however either why or how social support can reduce loneliness and psychological distress. Traditionally, research into the dynamics of support tends to examine sociological factors (such as age, gender, and social class), and individual-level variables, such as personality (66, 67). However, a meta-analysis showed weak–and at times inconsistent–evidence of the relationship between social support and health (68). Examining support from a social identity perspective provides a means to understand its social underpinnings, and from which to make sense of these contradictory findings.

According to the social identity approach, social connection provides a vehicle for accessing social support–both practically as well as psychologically, with both the receipt and provision of support known to be a resource harnessed through group membership (69–72). Of relevance here is a recent study with retirees (51). Steffens and colleagues' study examined the dual process of both support received and support provided to others. In line with evidence that feeling supported was beneficial for a range of outcomes [such as life satisfaction, subjective wellbeing, and improved physical health; (24, 27, 73, 74)], Steffens and colleagues found that feeling supported predicted wellbeing among retirees. Interestingly though, it was provision of support *to* others that more strongly explained the relationship from social connection to wellbeing. The latter finding is consistent with studies showing how providing help is associated with increased coping mechanisms, elevated feelings of life satisfaction, improved wellbeing (27, 75–79). Further, providing support to others has also been shown to decrease loneliness (26, 80), and these findings have been replicated across cultures (81).

Of relevance to the current research, the pathway from social connection to wellbeing has been shown to emerge under conditions of collective threat, such as public emergencies or natural disasters. Here we see that the perception of a common fate allows for the establishment of a shared identity, and that this emergent social identity leads to mutual support and subsequently to enhanced individual and collective health (82–84). Furthermore, research into formal support provision has demonstrated that rates of volunteerism are associated with increased feelings of personal self-efficacy and empowerment, as well as improved mental and physical health (85, 86), and that increases in shared identity are associated with higher levels of wellbeing for volunteers (87).

Building on the reasoning that social connectedness and social identification with groups unlocks psychological resources, a lack of social connectedness (i.e., loneliness), would prevent the action that would allow one to draw from those psychological resources. That is, higher levels of social isolation restrict the pathways–both logistical and psychological–that would allow individuals to draw effectively from social support. Consistent with this reasoning, higher loneliness has been found to be associated with lower levels of received social support (22, 23). Likewise, loneliness–and the lack of shared identity and connection with others that lies behind loneliness–limits the extent to which lonely individuals are motivated to provide social support to others. Supporting this, research has demonstrated links between increased loneliness and a reduction in pro-social tendencies, which includes a range of acts that are categorized as beneficial to others, including the enactment of support (88–92).

### Receipt and provision of social support during the COVID-19 pandemic

The pandemic provided a unique context for examining the relationships between social disconnection, support, and psychological distress. Under conditions of COVID-19, loneliness rates were elevated whilst the need for support as well as the need to help others was highly salient (13, 76, 93). Data collected during the pandemic demonstrated that, despite restrictions, people still endeavored to support each other (94), with evidence from across the globe of volunteering and the emergence of community-based mutual aid groups (95–97). Despite some acts of support making the headlines [such as the *Clap for our Carers* movement in the UK, (98)], the vast majority occurred at more local levels, and involved shopping, dog-walking, and other forms of emotional, informational or logistical support (99).

**Figure 1:**
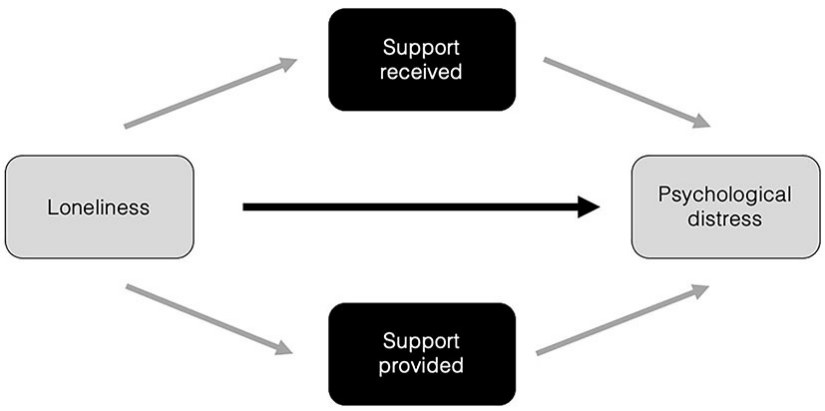
Hypothesized model showing the relationship between loneliness and psychological distress as mediated through both support received, and support provided.

Whilst there are established links between social support and wellbeing, as well as evidence that this relationship emerges more strongly as a result of a shared identity, less is known about this process during a crisis in which social connection (and the establishment of shared identity) was restricted. Within the context of the COVID-19 pandemic, we predict that a lack of social connection–and the risk of increased loneliness that arises from this–inhibited the process of both receiving help from others, as well as being able to provide support to others (32, 52, 71). Research would suggest that this inability to enact support is likely to exacerbate the relationship between loneliness and negative health outcomes, such as psychological distress. These relationships are illustrated in the model below (Figure 1), and which provides the basis for the hypotheses the present research tests.

## The present research

The COVID-19 pandemic created the context for a potential double jeopardy: social restrictions put people at risk of increased loneliness *and* reduced their capacity to engage pro-socially with others (to support others and benefit from support provided). We argue that social support, received and given, lies at the heart of this double jeopardy, offering a means to overcome the effect of loneliness on psychological distress. To test this relationship, we used a multi-country dataset to explore relationships between loneliness and psychological distress, with social support as a possible mediator. We first examined the more established route *via* feeling supported by others, and then examined the lesser-known pathway through provision of support *to* others.

Within this study there were two waves of data, both collected during the pandemic (March and June 2020; for a description of the pandemic conditions of each country at the time of data collection, refer to Supplementary material). In the first wave, residents from eleven countries took part in the study, providing a sample of 6,842 participants. Three months later we collected a second wave of data in three countries–the UK, Australia, and the US–surveying 1,299 of the same participants. Using the first wave of data, we first examined the relationships between loneliness, psychological distress, and social support, focusing particularly on the hypothesized mediating role of social support in the relationship between loneliness and psychological distress. Using the second wave of data, we explored these same relationships longitudinally. We expected to find that at Wave 1 higher levels of loneliness would be related to higher levels of psychological distress (H1a), and that higher levels of support received would be associated with lower levels of both loneliness and psychological distress (H1b). Further, we examined support provision, hypothesizing that this too would be associated with lower levels of both loneliness and psychological distress (H1c). We also expected to find that both forms of social support would mediate the relationship between loneliness and distress. Specifically, we hypothesized that lonelier people would feel less supported by others, which would in turn exacerbate their distress (H2a). We also hypothesized the lesser-known pathway through support provision, predicting that lonelier people would report less provision of support to others, which would in turn exacerbate their distress (H2b).

We also examined the direction of these relationships as they changed during the peak months of the pandemic. Here, we expected to find that increased loneliness (from Wave 1 to Wave 2) would lead to greater psychological distress (H3). Further, we expected receipt of social support from others (H4a) as well as provision of social support to others (H4b) to mediate this relationship over time, such that those reporting greater loneliness would see a decline in both forms of support, which would explain increases in psychological distress.

## Method

### Participants

A total of 6,842 participants were sampled across eleven countries: Australia, China, France, Germany, Indonesia, Italy, the Netherlands, Spain, Thailand, the UK and the US. Participants were sampled *via* either the Prolific crowd sourcing platform (Germany, Italy, the Netherlands, Spain, the United Kingdom and the United States), recruited through social media (Thailand and Indonesia), or both platforms (France and Australia). Countries were selected to represent as broad a dataset as possible but were also dictated by logistical constraints such as researcher access and funding restrictions. This first wave of data was collected during the pandemic in March 2020. For a description of the pandemic conditions of each country at the time of data collection, refer to Supplementary material.

In this Wave 1 sample, 532 participants (7.78%) were excluded after having failed an attention check (“To ensure you are a real human-being (and not a bot), please select strongly agree for this item”), leaving a final sample of 6,310 (54.90% identified as female; 43.60% male, 1.50% non-binary/other; *M*_age_ = 30.44, *SD* = 11.95). Additional sample characteristics for each country are presented in Supplementary Table 1. A Monte Carlo sensitivity power analysis for indirect effects (100) indicated that our final sample size in Wave 1 (*N* = 6,310) had 99% statistical power (a = 0.05) based on the strength of associations between our predictor, mediator, and outcome variables.

To provide us with longitudinal data, a smaller subset of this Wave 1 sample participated in a second survey undertaken 3 months later. Within this second wave, for reasons of convenience, data were collected from only three of the eleven countries, and comprised responses from 1,299 participants residing in Australia (*n* = 468), the US (*n* = 373) and the UK (*n* = 469). A total of 32 participants were excluded from Wave 2 after having failed an attention check, leaving a final sample of 1,267 (53.40% female; 45.50% male, 1.10% non-binary/other; *M*_age_ = 35.04, *SD* = 12.36). Additional sample characteristics for each country are presented in Supplementary Table 1. A Monte Carlo sensitivity power analysis for indirect effects (100) indicated that the final sample had 99% statistical power (a = 0.05) based on the strength of associations between our predictor, mediator, and outcome variables.

### Procedure

The study received ethical clearance *via* its university Ethics Committee (clearance number 2020000485). For Wave 1, data was collected between March 17^th^ and April 10^th^, 2020, and for Wave 2 between June 24^th^ and July 2^nd^, 2020. Surveys conducted in Chinese, Dutch, French, German, Indonesian, Italian, Thai, and Spanish were translated from English by the authors (all native speakers in their respective languages). Participant data collected on Prolific was advertised as a study looking at the effects of COVID-19 on people's thoughts and behavior,^1^ and participants were paid according to the platform's best practice guidelines. Once the participants had read a brief introduction to the study and were informed of their data anonymity and right to withdraw, they were asked for consent to proceed. Upon consent, participants were redirected to the survey which took approximately 15 min to complete.

### Measures

#### Loneliness

Loneliness was measured using four items adapted from Hughes (101); “I feel I lack companionship,” “I feel left out,” “I feel isolated from others,” and “I feel lonely;” α = 0.84. Participants were asked “How often do you feel like this in general?” and provided their responses to each statement using a scale from 1 (*Hardly ever*) to 3 (*Often*).

#### Social support received

Social support received was measured with three items (52): “I get the emotional support I need from other people,” “I get the help I need from other people,” “I get the resources I need from other people'; α = 0.87. Participants were asked to indicate their agreement using a scale ranging from 1 (*Strongly disagree*) to 7 (*Strongly agree)*.

#### Social support provided

Provision of support was measured using three items from Haslam and colleagues (52); “I give other people the emotional support they need,” “I give other people the help they need,” and “I give other people the resources they need;” α = 0.86. Participants were asked “When you think about people who are in your life, how much do you agree or disagree with these statements?” and indicated their agreement using a scale ranging from 1 (*Strongly disagree*) to 7 (*Strongly agree*).

#### Psychological distress

The Kessler Psychological Distress (K6) scale was used to measure distress over the past 30 days (102, 103). The K6 scale was developed as a screener for serious mental illness and was designed to provide a tool able to bridge between community and clinical epidemiology. Participants responded to the six words presented (e.g., “nervous,” “hopeless”) and asked to rate their frequency of occurrence from 1 (*None of the time*) to 5 (*All of the time*) (α = 0.87).

## Results

### Cross sectional analysis

#### Descriptive data

Table 1 displays the overall means, standard deviations, and bivariate correlations of key variables collapsed across the eleven Wave 1 countries. Results for each of the eleven countries are presented individually in the Supplementary Table 2.

**Table 1:** Mean, standard deviation and bivariate correlations of key variables, wave 1.

**Variable**	***M***	***SD***	**Correlations**			
			**1**	**2**	**3**	**4**
1. Loneliness	1.58	0.56	-			
2. Social support provided	5.46	0.99	−0.18^**^	-		
3. Social support received	5.23	1.19	−0.34^**^	0.52^**^	-	
4. Psychological distress	2.02	0.81	0.54^**^	−0.13^**^	−0.26^**^	-

N = 6,310 ^**^p < 0.01.

### Multi-level mediation

To test our hypotheses, we conducted multi-level path analysis in MPlus version 8.3 (104) to account for clustering of the data (11 countries, total *N* = 6,842). The key difference between multi-level mediation and standard mediation is the presence of random intercepts (i.e., allowing the intercept within each country to vary) which allowed us to control for country-level differences. Since our hypotheses focused on individual-level variables (i.e., participant perceptions and experiences), we focused on the within-level mediation effects (Level 1) and used group-mean centering of the predictor variables to center the predictors within each country (105). We note that a small amount of the variance in psychological distress (ICC = 0.06) was attributable to country-level differences. All analyses controlled for participants' age and gender.

Using multi-level path analysis, we first tested the association between loneliness and psychological distress (H1a), and then the associations between social support (both receipt and provision) and loneliness and psychological distress (H1b and H1c). We found that loneliness was significantly associated with higher psychological distress [*b* = 0.52 (0.494, 0.544), *SE* = 0.01, *p* < 0.001], providing support for H1a. As expected, we found that receipt of support was significantly negatively associated with psychological distress [*b* = −0.28 (-0.320,−0.248), *SE* = 0.02, *p* < 0.001] and significantly negatively associated with loneliness [*b* = −0.34 (-0.388,−0.292), *SE* = 0.03, *p* < 0.001], providing support for H1b. At the same time, we found that this same relationship was observed for provision of social support, such that it was significantly negatively associated with psychological distress [*b* = −0.16 (-0.187,−0.131), *SE* = 0.02, *p* < 0.001] and significantly negatively associated with loneliness [*b* = −0.19 (-0.244,−0.143), *SE* = 0.03, *p* < 0.001], providing support for H1c.

**Figure 2:**
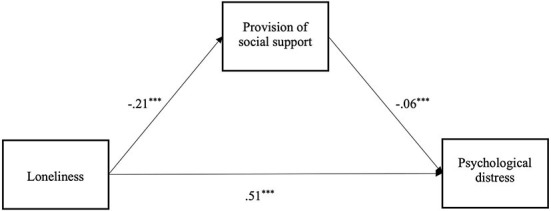
Wave 1 mediation model of the effect of loneliness on psychological distress, *via* social support received (***p < 0.001).

In a second step, we tested our mediation hypotheses (H2a and H2b) using both types of social support as mediators. Figure 2 shows the findings from multilevel analysis with the relationship between loneliness and psychological distress mediated by social support received. As expected, higher levels of loneliness predicted less social support received [*b* = −0.35 (-0.401,−0.289), *SE* = 0.03, *p* < 0.001], and less received social support in turn predicted higher levels of psychological distress [*b* = −0.13 (-0.162,−0.088), *SE* = 0.02, *p* < 0.001]. The indirect effect between loneliness and psychological distress via social support received was significant [*b* = 0.04 (0.027, 0.059), *SE* = 0.01, *p* < 0.001], providing support for H2a. After accounting for this indirect effect, the direct effect between loneliness and psychological distress remained significant [*b* = 0.48 (0.437, 0.513), *SE* = 0.02, *p* < 0.001].

**Figure 3:**
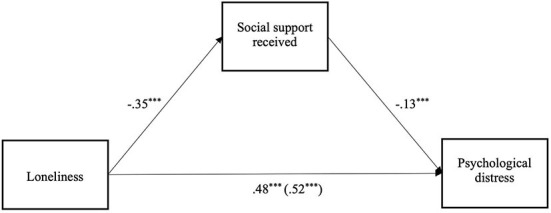
Wave 1 mediation model of the effect of loneliness on psychological distress, via provision of social support (***p < 0.001).

We then examined the alternative pathway through provision of social support. Figure 3 shows the findings from multilevel analysis with the relationship between loneliness and psychological distress mediated by provision of social support. As hypothesized, higher levels of loneliness predicted less provision of social support [*b* = −0.21 (-0.257,−0.156), *SE* = 0.03, *p* < 0.001], and less provision of social support in turn predicted higher levels of psychological distress [*b* = −0.06 (-0.080,−0.040), *SE* = 0.01, *p* < 0.001]. The indirect effect between loneliness and psychological distress *via* provision of social support was significant [*b* = 0.01 (0.007, 0.017), *SE* = 0.003, *p* < 0.001], providing preliminary support for H2b. After accounting for this indirect effect, the direct effect between loneliness and psychological distress remained significant [*b* = 0.51 (0.479, 0.535), *SE* = 0.01, *p* < 0.001].

### Longitudinal analysis

#### Descriptive results and correlations

Table 2 displays the overall means, standard deviations, and bivariate correlations of key variables collapsed across the three Wave 2 countries. Results showed a significant increase in loneliness from Wave 1 (*M* = 1.62) to Wave 2 (*M* = 1.68), *t*_(1, 263)_ = −5.08, *p* < 0.001, as the pandemic progressed. Furthermore, and in line with Wave 1 findings, loneliness was positively associated with psychological distress at both timepoints (H1a). On looking at social support–and again in line with Wave 1 findings–support received was significantly negatively associated with both loneliness and psychological distress at both timepoints (H1b), as was support provided (H1c).

**Table 2:** Means, standard deviations, and bivariate correlations for key variables wave 1 and 2.

**Variable**	***M***	***SD***	**Correlations**							
			**1**	**2**	**3**	**4**	**5**	**6**	**7**	**8**
1. Loneliness (wave 1)	1.62	0.58	-							
2. Social support provided (wave 1)	5.57	1.01	−0.28^**^	-						
3. Social support received (wave 1)	5.20	1.28	−0.42^**^	0.54^**^	-					
4. Psychological distress (wave 1)	2.05	0.84	0.52^**^	−0.18^**^	−0.31^**^	-				
5. Loneliness (wave 2)	1.68	0.62	0.68^**^	−0.18^**^	−0.35^**^	0.47^**^	-			
6. Social support provided (wave 2)	5.46	1.04	−0.25^**^	0.65^**^	0.40^**^	−0.17^**^	−0.22^**^	-		
7. Social support received (wave 2)	5.08	1.32	−0.40^**^	0.40^**^	0.66^**^	−0.30^**^	−0.47^**^	0.55^**^	-	
8. Psychological distress (wave 2)	1.99	0.87	0.45^**^	−0.17^**^	−0.29^**^	0.72^**^	0.57^**^	−0.19^**^	−0.41^**^	-

N = 1,267. ^**^p < 0.01.

### Longitudinal relationships

To explore changes in loneliness and distress at Wave 1 and Wave 2, we used SPSS (v28) to conduct a regression between loneliness at Wave 1 and psychological distress at Wave 2, controlling for psychological distress, age and gender at Wave 1. We found that loneliness at Wave 1 significantly predicted psychological distress at Wave 2 (*R*^2^ = 0.54, *F*_(4, 1, 259)_ = 364.960, *p* < 0.001); providing support for H3.

### Longitudinal mediation

To test H4a and H4b, we conducted mediation analysis using MPlus version 8.3 (104). The ICC showed that a very small amount of the variance in psychological distress was attributable to national differences (ICC < 0.01), which is consistent with the ICC of psychological distress at Wave 1. Therefore, we conducted the mediation with the collapsed data across three countries, but we note that the conclusions were identical when the same mediation was conducted through multilevel modeling while controlling for country-level differences.

**Figure 4:**
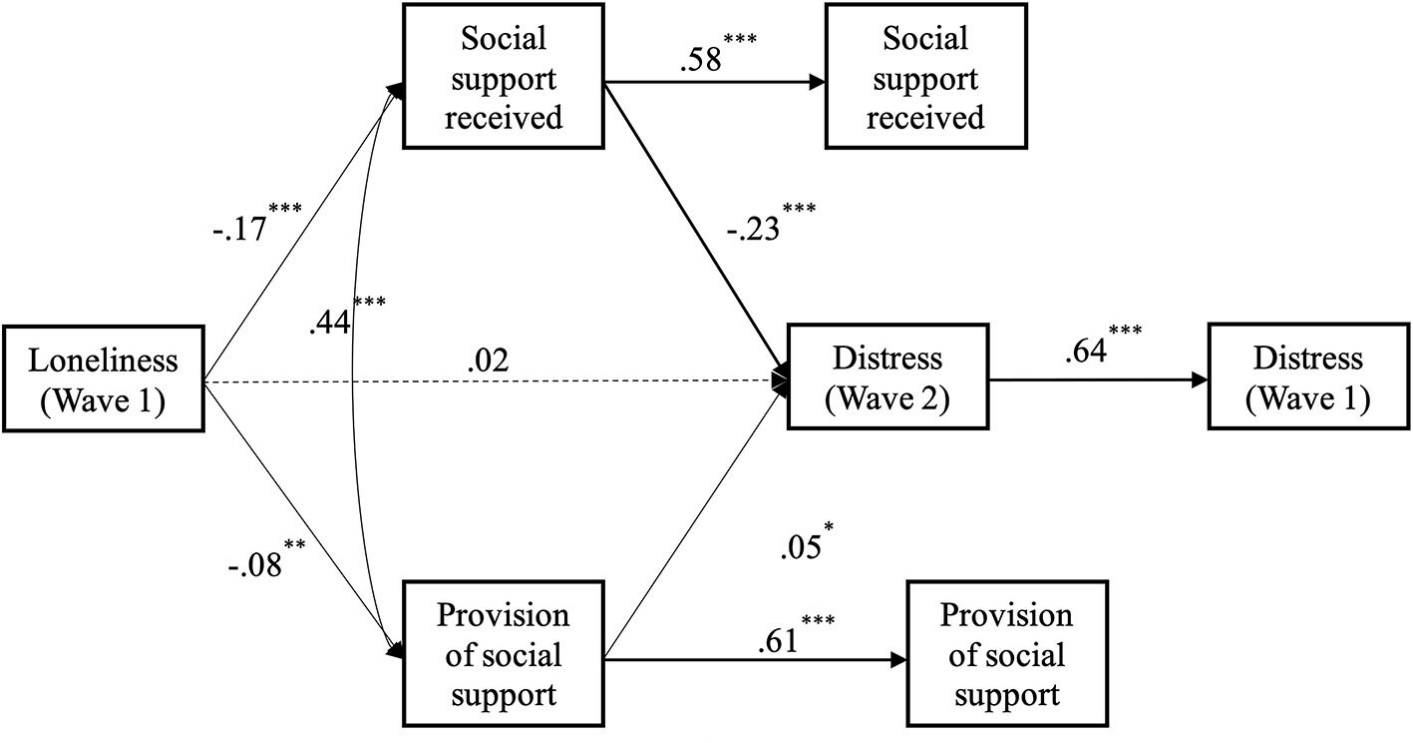
Longitudinal mediation model of the effect of loneliness at wave 1 on psychological distress at wave 2, *via* social support received at wave 2, while controlling for wave 1 levels of distress and social support received (* *p* < 0.05. **p < 0.01. ***p < 0.001).

Starting with support received (H4a), we found a longitudinal result whereby loneliness predicted increased psychological distress over time, and that this was mediated by social support received (see Figure 4). Specifically, greater loneliness at Wave 1, predicted reduced social support received at Wave 2 [*b* = −0.16 (-0.204,−0.106), *SE* = 0.03, *p* < 0.001], over and above Wave 1 social support received [*b* = 0.59 (0.542, 0.641), *SE* = 0.03, *p* < 0.001]. Reduced social support received at Wave 2 in turn predicted increased psychological distress at Wave 2 [*b* = −0.21 (-0.251,−0.160), *SE* = 0.02, *p* < 0.001], over and above Wave 1 distress [*b* = 0.64 (0.597, 0.687), *SE* = 0.02, *p* < 0.001]. The indirect effect between Wave 1 loneliness and Wave 2 psychological distress *via* Wave 2 social support received was significant [*b* = 0.03 (0.020, 0.044), *SE* = 0.01, *p* < 0.001]. After accounting for this indirect effect, the direct effect between Wave 1 loneliness and Wave 2 distress was not significant [*b* = 0.02 (-0.030, 0.065), *SE* = 0.02, *p* = 47], providing support for H4a and aligning with Wave 1 results.

**Figure 5:**
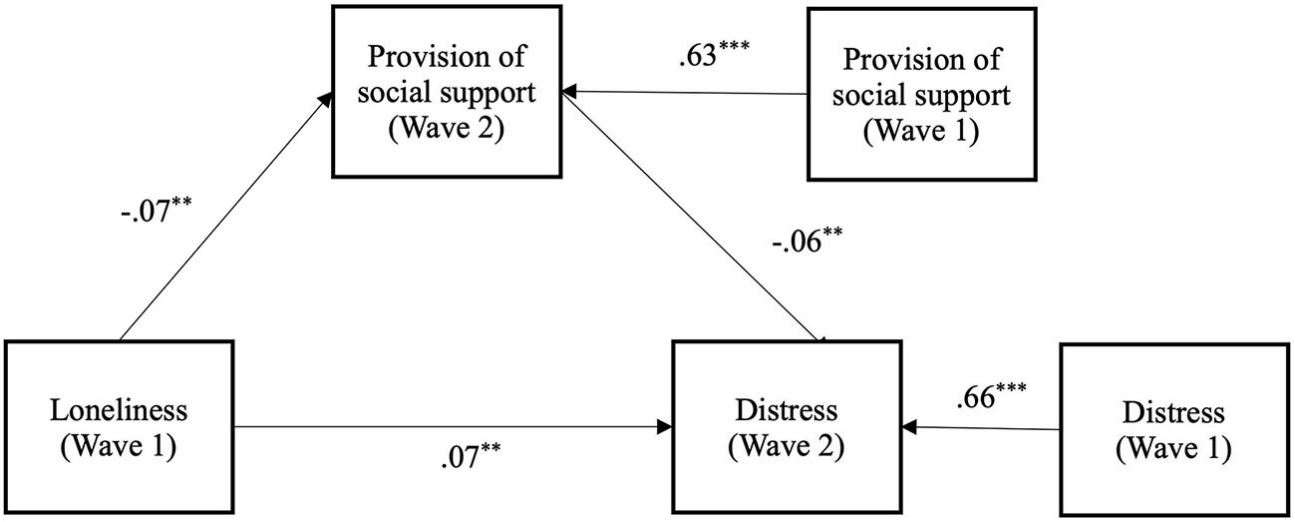
Longitudinal mediation model of the effect of loneliness at wave 1 on psychological distress at wave 2, *via* provision of social support at wave 2, while controlling for wave 1 levels of distress and provision of social (** *p* < 0.01. ***p < 0.001).

As previously analyzed with the Wave 2 data, we also examined the alternative pathway of providing support to others (H4b). Here, we found a longitudinal link whereby loneliness predicted increased psychological distress over time, and that this was again mediated by the provision of social support (see Figure 5). Specifically, greater loneliness at Wave 1, predicted reduced provision of social support at Wave 2 [*b* = −0.07 (-0.117,−0.027), *SE* = 0.02, *p* < 0.01], over and above Wave 1 provision of social support [*b* = 0.63 (0.579, 0.677), *SE* = 0.03, *p* < 0.001]. Reduced provision of social support at Wave 2 in turn predicted increased psychological distress at Wave 2 [*b* = −0.06 (-0.100,−0.022)], *SE* = 0.02, *p* < 0.01], over and above Wave 1 distress [*b* = 0.66 (0.619, 0.707), *SE* = 0.02, *p* < 0.001]. The indirect effect between Wave 1 loneliness and Wave 2 psychological distress *via* Wave 2 provision of social support was significant [*b* = 0.004 (0.000, 0.008), *SE* = 0.002, *p* < 0.05]; providing support for H4b. After accounting for this indirect effect, the direct effect between Wave 1 loneliness and Wave 2 distress was still significant [*b* = 0.07 (0.025, 0.120), *SE* = 0.02, *p* < 0.01].

**Figure 6:**
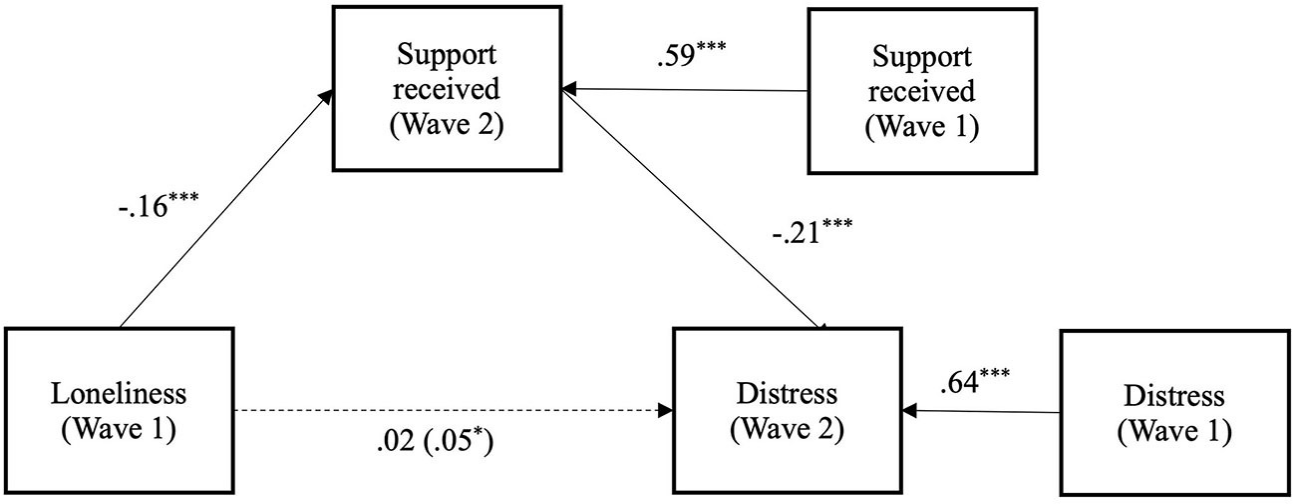
Longitudinal mediation model of the effect of loneliness at wave 1 on psychological distress at wave 2, *via* social support received at wave 2 and provision of social support at wave 2, while controlling for wave 1 levels of distress, provision of social support, and social Support Received (* *p* < 0.05. ** *p* < 0.01. ***p < 0.001).

A final analysis examined the effects of both provided and received support simultaneously as mediators of the relationship between loneliness and distress (see Figure 6). We found that greater loneliness at Wave 1, predicted reduced social support received at Wave 2 [*b* = −0.17 (-0.215,−0.119), *SE* = 0.02, *p* < 0.001], over and above Wave 1 social support received [*b* = 0.58 (0.525, 0.624), *SE* = 0.03, *p* < 0.001]. At the same time, reduced social support received at Wave 2 predicted increased psychological distress at Wave 2 [*b* = −0.23 (-0.284,−0.173), *SE* = 0.03, *p* < 0.001], over and above Wave 1 distress [*b* = 0.64 (0.596, 0.686), *SE* = 0.02, *p* < 0.001]. The indirect effect between Wave 1 loneliness and Wave 2 psychological distress *via* Wave 2 received social support was significant [*b* = 0.04 (0.024, 0.053), *SE* = 0.01, *p* < 0.001]. We also found that greater loneliness at Wave 1 predicted reduced provision of social support at Wave 2 [*b* = −0.08 (-0.125,−0.034), *SE* = 0.02, *p* < 0.01], over and above Wave 1 provision of social support [*b* = 0.61 (0.565, 0.662), *SE* = 0.03, *p* < 0.001]. Reduced provision of social support at Wave 2 predicted reduced Wave 2 psychological distress [*b* = 0.05 (0.002, 0.095), *SE* = 0.02, *p* < 0.05], over and above Wave 1 distress [*b* = 0.64 (0.596, 0.686), *SE* = 0.02, *p* < 0.001]. The indirect effect between Wave 1 loneliness and Wave 2 psychological distress *via* Wave 2 provision of social support was not significant [*b* = −0.004 (-0.008, 0.000), *SE* = 0.002, *p* = 0.079]. After accounting for both indirect effects, the direct effect between Wave 1 loneliness and Wave 2 distress was not significant [*b* = 0.02 (-0.028, 0.067), *SE* = 0.02, *p* = 0.43].

## Discussion

During the COVID-19 pandemic levels of loneliness and psychological distress increased across the world (8, 9, 11). There were many contributing factors to these outcomes, not least, a significant reduction in the richness of social interaction that was enforced to curb the spread of the virus (4, 10, 106). Social distancing requirements and the various stay-at-home orders also meant that people's ability to receive support and support others–key hypothesized mechanisms through which to both overcome loneliness and associated psychological distress–was hindered. Enforced social isolation therefore not only reduced people's ability to enact social connection, but this is turn made social support a challenge–both logistically and psychologically, thereby creating a double jeopardy. In our research, we examined the role played by social support in the relationship between loneliness and psychological distress, particularly testing the pathway from loneliness to distress *via* both received and provided social support. The latter pathway *via* support provision is currently under-investigated in the literature but was particularly relevant during the COVID-19 pandemic in which providing support to more vulnerable groups became highly salient (11, 17–20).

We interrogated a large multi-county dataset across eleven countries and found a significant association between loneliness and distress. Using longitudinal data from three countries, we found evidence that this relationship unfolded over time in the hypothesized direction such that increased loneliness predicted increased distress. This extends the loneliness literature by providing evidence for directionality in this relationship from loneliness to distress (12, 107). We also found that increased social support–both received and provided–was associated with lower levels of loneliness and psychological distress. Specifically, across time, we found that a sense of both being supported as well as providing support for others partially explained the relationship between loneliness and psychological distress, such that lonelier people reported lower levels support receipt and provision, and this in turn caused them more psychological distress. These longitudinal findings confirm the positive role played by support given and received in the relationship between loneliness and distress (21–23, 64, 65).

The more novel demonstration of the importance of support provision provides further evidence of how helping others can mediate the relationship between group connectedness and improved health and wellbeing (51, 52, 71). Of note however, when examining both forms of support together, it appeared that receiving support had more impact on the relationship between loneliness and distress than provision of support. This might be a reflection of the difficulties people had in providing support to others due to enforced social distancing measures. It might also be a reflection of the importance of feeling supported *by others* through a highly stressful event, and which fits with other data collected during the pandemic demonstrating the relationship between lower levels of support received and psychological distress (108).

The current findings have several theoretical implications. Using a large, multi-country dataset, our results provide an empirically tested model of the directional relationship between loneliness and distress during COVID-19. Further, our results highlight a key underlying mechanism–that of social support. Social support has previously been shown to play a key role in unlocking the social cure benefits of group connectedness (52, 71). At the same time, the enactment of support provides a means of structuring and cementing social connection (33, 109). This aligns with previous research that has shown how social support is associated with improved wellbeing and reduced loneliness (26, 27, 75, 78, 79).

The current research extends our understanding of these findings by demonstrating that benefits of support flow two ways–both feeling supported and feeling more able to support others reduces psychological distress. What we also found was that people who felt lonelier were less likely to be able to activate and engage in support receipt and support provision. Demonstrating this relationship at a time when social connection opportunities were restricted is particularly important as many people–but especially more vulnerable groups–were at a heightened risk of increased levels of loneliness, and thus more vulnerable to increased psychological distress (11, 13, 17–20).

In addition to the theoretical implications noted above, the practical implications of these findings inform our understanding of how to reduce loneliness and psychological distress in the event of future public challenges, particularly ones associated with increased social disconnection. Social support is already recognized as a key factor in the management of public health (62, 63). However, in addition to targeting receipt of social support, governments, organizations, and communities, could benefit from investing in policies and procedures to direct, scaffold, and promote opportunities to create an increased sense of social connection, particularly through providing the means for people to engage in all forms of social support. This may take the form of educational material in which the importance of group connectedness can be promoted (see GROUPS 2 CONNECT; 106), or could be established through financial or structural support for the creation and maintenance of community-based mutual aid groups (95). Enactment of support would thus create a platform for the establishment of social connection and for harnessing the measurable benefits of a social cure. Beyond COVID-19 or similar events, a greater understanding of the power of social support might also benefit the management of what has recently been referred to as the loneliness epidemic (54, 110–112).

### Strengths, limitations, and future directions

This study analyzed data from eleven different countries across the globe. Such a large dataset provided us with a significant source of information with which to understand loneliness, psychological distress, and provision of social support. However, a limitation of using crowd sourcing platforms, as well as convenience samples for some countries, was that the sample is unlikely be fully representative. A further limitation was that the measures used were deliberately brief due to the data forming part of a much larger survey. As such, it would have been preferable to have more measures with which to validate the constructs of interest, using clinical measures of other related constructs such as depression or anxiety. Methodological limitations also resulted from the cross-sectional nature of the Wave 1 data. However, being able to test the same analysis longitudinally through inclusion of the Wave 2 data, did strengthen our analysis. It is worth noting however that within the multi-level model, the co-efficient from social support to psychological distress was small (but significant). Future research using alternative datasets collected during the COVID-19 pandemic in which the same, or similar variables were measured, could shed more light on the extent of these relationships.

## Conclusion

Dealing with crisis events such as the COVID-19 pandemic requires the management of both structural issues, and the related psychological fall-out caused by social disconnection and loneliness. The current research contributes to our understanding of factors that might mitigate the negative outcomes associated with these conditions. We showed that increased loneliness led to psychological distress, and that this relationship could be explained by both perceived feelings of being unsupported, as well as feeling unable to support others. In times of crisis, providing a means for people to take more positive social action–to help others–has the advantage of providing support for those in need as well as delivering a social cure for those giving support.

## Data availability statement

The raw data supporting the conclusions of this article will be made available by the authors, without undue reservation.

## Ethics statement

The studies involving human participants were reviewed and approved by School of Psychology, The University of Queensland. The participants provided their written informed consent to participate in this study.

## Author contributions

SB, TY, BA, JJ, and CH contributed to conception and design of the study. BA organized the database and performed the statistical analysis. SB wrote the first draft of the manuscript. All authors contributed to manuscript revision, read, and approved the submitted version.

## Funding

Work on this paper was supported by an Australian Research Council Laureate Fellowship FL110100199 awarded to the JJ.

## Conflict of interest

The authors declare that the research was conducted in the absence of any commercial or financial relationships that could be construed as a potential conflict of interest.

## Publisher's note

All claims expressed in this article are solely those of the authors and do not necessarily represent those of their affiliated organizations, or those of the publisher, the editors and the reviewers. Any product that may be evaluated in this article, or claim that may be made by its manufacturer, is not guaranteed or endorsed by the publisher.

## Footnotes

^1^The data reported in this study formed part of a wider survey looking into a range of other issues related to the experience of living through the COVID-19 pandemic. For information on other variables measured: https://osf.io/6t3y7/.
