# Supplementary material for: Double jeopardy: How lower levels of support during COVID-19 exacerbated the relationship between loneliness and distress
Source: Front Public Health. 2022 Aug 26;10:976443. doi: 10.3389/fpubh.2022.976443 (PMC9459340; doi:10.3389/fpubh.2022.976443)
Supplement: Supplementary file 1 [file Data_Sheet_1.PDF]

Supplementary material

Table S1

Contextual information regarding the cross-national samples for Wave 1 and 2

Country	Date of data collection (in 2020)	Contextual information
Australia<br>*n* = 1,480	March 17-20	There were 455 cases on March 17 and 928 on March 20 (14 deaths). Most flights were cancelled on the 19th; indoor gatherings of > 100 people were banned; supermarkets tightened food buying restrictions.
U.K.<br>*n* = 535	March 24	Total number of confirmed cases was 8,200, with 1,400 new cases that day (deaths = 422). British government ordered a lockdown on March 23.
USA<br>*n* = 496	March 25	Total number of cases was 66,790, with 13,355 new cases that day. Total deaths by that date was 1,027. Many states were closing businesses, schools, and workplaces, and in many cases, citizens were requested to stay home.
Italy<br>*n* = 498	March 27-28	Total number of cases on March 27 was 86,500 and 92,500 on March 28. Total number of deaths on March 27 were 9,134 rising to 10,023 on March 28. Italy issued a nationwide lockdown on March 9, which was still in place at the time of data collection.
Germany<br>*n* = 498	March 26-27	Number of cases was 36,500 on March 26 and 42,288 on March 27. Number of deaths was 198 on March 26 rising to 253 on March 27. Strict social distancing measures were issued on March 22 and were still in place at the time of data collection.
Netherlands<br>*n* = 403	March 30 – April 4	By March 30 there were 11,750 total cases, and 864 deaths. By April 4th the total number of cases were 16,627 and 1,651 deaths. New cases were going down between March 30 and April 4 (from 1,200 to 780). Employees of non-essential jobs were asked to work from home: bars, restaurants, museums, schools and universities were closed, as were public gatherings and large-scale events.
Spain<br>*n* = 489	March 31 – April 1	By March 31 they were 94,417 cases, 8,189 deaths and 49,243 people in hospitals. New cases and deaths were still increasing. Spain went into mandatory lockdown on March 15, which was extended for an additional 2 weeks on March 26th.

France<br>*n* = 523	March 27 – April 7	By March 27 there were 32,964 cases and 1,995 deaths. By April 7 the total number of cases were 109,069 and 10,328 deaths. France was in a strict nationwide lockdown during the time of data collection.
China<br>*n* = 611	March 26 – April 2	By March 26 they were 81.394 total cases, and 3.295 total deaths. By April 2 the total number of cases were 81.620 and 3.322 deaths. China started to “flatten the curve” around February 15, so by the time of data collection the situation was “controlled” and there was a lot of news around some restriction being lifted in Wuhan. By March 30, Wuhan reported little increase in cases. Some lockdown restrictions were lifted during the first weeks of April, after almost 3 months of lockdown (in Wuhan). Restrictions will be lifted gradually, and many people are still in lockdown.
Indonesia<br>*n* = 464	March 25 – April 9	By March 25 they were 790 total cases, and 58 total deaths. By April 9 the total number of cases were 3.293 and 280 deaths. During the days of data collection, the number of cases started to go exponentially up, and they have yet to reach the peak of the curve. Indonesia has the higher number of deaths in Asia after China. Until early March, the government claimed it had no cases of infection, and here are indications that many cases and deaths are going undetected due to insufficient testing. There was no lockdown at the time of data collection, on March 31st a state of emergency was declared with measures of social distancing, and on the week of April 6th, social distancing measures were stricter, closing offices, schools, and ban of gathering of more than five people in Jakarta
Thailand<br>*n* = 313	March 30 – April 10	Data was collected between March 30st and April 10. By March 30 they were 1524 total cases, and 9 total deaths. By April 10 the total number of cases were 2.473 and 33 deaths. During the days of data collection, the number of new cases per day started to go down, and they entered a phase of a ‘flat curve’. Thailand was on partial lockdown at the time of data collection. A state of emergency was in place, and an entry ban to the country. On March 27, Thailand went into broad lockdown when the state of emergency was announced a few days before. Borders were closed to international visitors, social gatherings were banned

		and closed facilities (libraries, museums, playgrounds, etc.) until the end of April. On March 30, Phuket Island went into a lockdown.
Australia (Wave 2), *n* =	June 24 – July 2	At the time of data collection, a second wave of cases was starting (specifically in the state of Victoria) with cases increasing (29 cases on June 24 and 81 on July 2). There was no lockdown until July 2, when around 30,000 people living in the state of Victoria received stay-at- home orders
UK (Wave 2), *n* =	June 25 – July 1	At the time of data collection, cases were variable (652 new cases on June 24, 1,380 on June 27, and 689 on July 2). The UK was preparing for the easing of more lockdown restrictions that were going to start on July 4 (reopening of restaurants, pubs, hotels and hairdressers.
USA (Wave 2), *n* =	June 24 – July 2	At the time of data collection, cases were still increasing (37,945 new cases on June 24 and 54,869 on July 2). Staying at home or social distancing restrictions varied by state, county, and/or city. Black Lives Matter protests after George Floyd’s murder started about a month before data collection (on May 30).

Table S2

Means, standard deviations and correlations between key variables broken down by country, Wave 1

	M	*SD*	Range	Correlations			
				1	2	3	4
**Australia**							
1. Loneliness	1.59	0.56	1-3	-			
2. Provision of social support	5.61	0.96	1-7	-.30**	-		
3. Social support received	5.28	1.25	1-7	-.43**	.53**	-	
4. Psychological distress	2.00	0.81	1-5	.53**	-.20**	-.32**	-
**China**							
1. Loneliness	1.37	0.46	1-3	-			
2. Provision of social support	5.06	0.91	1-7	-.13**	-		
3. Social support received	5.01	0.96	1-7	-.11**	.67**	-	
4. Psychological distress	1.52	0.53	1-5	.53**	-.14**	-.10*	-
**France**							
1. Loneliness	1.65	0.59	1-3	-			
2. Provision of social support	5.41	0.95	1-7	-.16**	-		
3. Social support received	5.20	1.33	1-7	-.31**	.41**	-	
4. Psychological distress	2.02	0.79	1-5	.52**	-.20**	-.28**	-
**Germany**							
1. Loneliness	1.60	0.52	1-3	-			
2. Provision of social support	5.35	1.05	1-7	-.18**	-		
3. Social support received	5.19	1.18	1-7	-.37**	.56**	-	
4. Psychological distress	1.99	0.77	1-5	.53**	-.14**	-.41**	-
**Indonesia**							
1. Loneliness	1.58	0.52	1-3	-			
2. Provision of social support	5.50	0.89	1-7	-0.03	-		
3. Social support received	5.41	1.00	1-7	-.23**	.45**	-	
4. Psychological distress	2.30	0.75	1-5	.56**	0.00	-.13**	-
**Italy**							
1. Loneliness	1.78	0.58	1-3	-			
2. Provision of social support	5.43	0.98	1-7	-.14**	-		

3. Social support received	5.08	1.24	1-7	-.26**	.38**	-	
4. Psychological distress	2.16	0.85	1-5	.53**	-.17**	-.32**	-
**Netherlands**							
1. Loneliness	1.55	0.55	1-3	-			
2. Provision of social support	5.43	0.95	1-7	-.24**	-		
3. Social support received	5.39	1.06	1-7	-.31**	.41**	-	
4. Psychological distress	1.96	0.74	1-5	.52**	-.20**	-.28**	-
**Spain**							
1. Loneliness	1.52	0.54	1-3	-			
2. Provision of social support	5.58	1.05	1-7	-.23**	-		
3. Social support received	5.42	1.16	1-7	-.35**	.64**	-	
4. Psychological distress	2.08	0.76	1-5	.50**	-.13**	-.22**	-
**Thailand**							
1. Loneliness	1.40	0.49	1-3	-			
2. Provision of social support	5.21	0.94	1-7	-.13*	-		
3. Social support received	5.17	1.06	1-7	-0.11	.46**	-	
4. Psychological distress	2.14	0.79	1-5	.61**	-.15**	-0.06	-
**United Kingdom**							
1. Loneliness	1.59	0.57	1-3	-			
2. Provision of social support	5.80	0.86	1-7	-.24**	-		
3. Social support received	5.38	1.19	1-7	-.42**	.52**	-	
4. Psychological distress	2.09	0.85	1-5	.56**	-.17**	-.33**	-
**United States**							
1. Loneliness	1.71	0.61	1-3	-			
2. Provision of social support	5.38	1.13	1-7	-.27**	-		
3. Social support received	4.99	1.40	1-7	-.44**	.50**	-	
4. Psychological distress	2.20	0.90	1-5	.52**	-.17**	-.36**	-

* p < .05 ** p < .01

Table S3

Means, standard deviations and correlations between key variables broken down by country, Wave 2

	M	*SD*	Correlations							
			1	2	3	4	5	6	7	8
**Australia**										
1. Loneliness (wave 1)	1.59	0.56	-							
2. Provision of social support (wave 1)	5.48	1.00	-.30**	-						
3. Social support received (wave 1)	5.13	1.27	-.39**	.53**	-					
4. Distress (wave 1)	1.99	0.83	.48**	-.21**	-.28**	-				
5. Loneliness (wave 2)	1.71	0.61	.66**	-.22**	-.32**	.46**	-			
6. Provision of social support (wave 2)	5.36	1.01	-.27**	.59**	.41**	-.20**	-.22**	-		
7. Social support received (wave 2)	4.93	1.32	-.36**	.34**	.63**	-.24**	-.45**	.54**	-	
8. Distress (wave 2)	2.00	0.87	.37**	-.18**	-.21**	.70**	.54**	-.21**	-.35**	-
**UK**										
1. Loneliness (wave 1)	1.59	0.56	-							
2. Provision of social support (wave 1)	5.82	0.85	-.23**	-						
3. Social support received (wave 1)	5.39	1.17	-.41**	.52**	-					
4. Distress (wave 1)	2.07	0.84	.55**	-.16**	-.34**	-				
5. Loneliness (wave 2)	1.63	0.62	.67**	-.10*	-.36**	.47**	-			
6. Provision of social support (wave 2)	5.65	0.96	-.19**	.63**	.37**	-.12**	-.15**	-		
7. Social support received (wave 2)	5.31	1.23	-.36**	.38**	.62**	-.35**	-.46**	.55**	-	
8. Distress (wave 2)	1.95	0.86	.47**	-0.09	-.30**	.73**	.59**	-.11*	-.44**	-

**US**										
1. Loneliness (wave 1)	1.68	0.60	-							
2. Provision of social support (wave 1)	5.36	1.12	-.31**	-						
3. Social support received (wave 1)	5.05	1.39	-.46**	.54**	-					
4. Distress (wave 1)	2.09	0.85	.53**	-.18**	-.33**	-				
5. Loneliness (wave 2)	1.71	0.61	.71**	-.20**	-.35**	.50**	-			
6. Provision of social support (wave 2)	5.32	1.14	-.29**	.70**	.40**	-.19**	-.27**	-		
7. Social support received (wave 2)	4.98	1.38	-.50**	.44**	.70**	-.36**	-.50**	.53**	-	
8. Distress (wave 2)	2.04	0.87	.50**	-.23**	-.35**	.75**	.56**	-.23**	-.44**	-
